# Family model of HIV care and treatment: a retrospective study in Kenya

**DOI:** 10.1186/1758-2652-15-8

**Published:** 2012-02-22

**Authors:** Jayne Lewis Kulzer, Jeremy A Penner, Reson Marima, Patrick Oyaro, Arbogast O Oyanga, Starley B Shade, Cinthia C Blat, Lennah Nyabiage, Christina W Mwachari, Hellen C Muttai, Elizabeth A Bukusi, Craig R Cohen

**Affiliations:** 1 Family AIDS Care and Education Services (FACES), Research Care and Training Program, Centre for Microbiology Research, Kenya Medical Research Institute, Kisumu, Kenya; 2 Department of Obstetrics, Gynecology and Reproductive Sciences, University of California San Francisco, San Francisco, CA, USA; 3 Department of Family Practice, University of British Columbia, Vancouver, BC, Canada; 4 Department of Medicine, University of California San Francisco, San Francisco, CA, USA; 5 Provincial AIDS/STD Control Operations, Kenya Ministry of Health, Nairobi, Kenya; 6 Centre for Respiratory Disease Research, Kenya Medical Research Institute, Nairobi, Kenya; 7 Division of Global HIV/AIDS, Centers for Disease Control and Prevention, Kisumu, Kenya; 8 Department of Obstetrics, Gynecology and Reproductive Sciences, University of California San Francisco, 50 Beale St., Suite 1200, San Francisco, CA 94105, USA

## Abstract

**Background:**

Nyanza Province, Kenya, had the highest HIV prevalence in the country at 14.9% in 2007, more than twice the national HIV prevalence of 7.1%. Only 16% of HIV-infected adults in the country accurately knew their HIV status. Targeted strategies to reach and test individuals are urgently needed to curb the HIV epidemic. The family unit is one important portal.

**Methods:**

A family model of care was designed to build on the strengths of Kenyan families. Providers use a family information table (FIT) to guide index patients through the steps of identifying family members at HIV risk, address disclosure, facilitate family testing, and work to enrol HIV-positive members and to prevent new infections. Comprehensive family-centred clinical services are built around these steps. To assess the approach, a retrospective study of patients receiving HIV care between September 2007 and September 2009 at Lumumba Health Centre in Kisumu was conducted. A random sample of FITs was examined to assess family reach.

**Results:**

Through the family model of care, for each index patient, approximately 2.5 family members at risk were identified and 1.6 family members were tested. The approach was instrumental in reaching children; 61% of family members identified and tested were children. The approach also led to identifying and enrolling a high proportion of HIV- positive partners among those tested: 71% and 89%, respectively.

**Conclusions:**

The family model of care is a feasible approach to broaden HIV case detection and service reach. The approach can be adapted for the local context and should continue to utilize index patient linkages, FIT adaption, and innovative methods to package services for families in a manner that builds on family support and enhances patient care and prevention efforts. Further efforts are needed to increase family member engagement.

## Background

Nyanza Province had the highest HIV prevalence in Kenya at 14.9% in 2007, more than twice the national HIV prevalence of 7.1% [1]. Despite this high HIV prevalence, the majority of Kenyans were unaware of their status: only one-third of adults had been tested for HIV and just 16% of HIV-infected adults accurately knew their HIV status [1].

Stigma, denial and fear of rejection continue to impede HIV testing, and along with limited access to care and treatment services, act as barriers to engaging in medical care for those who test HIV positive [2-5]. Children are particularly vulnerable to HIV infection if their mothers are HIV-infected, and their HIV status often goes undetected; there are an estimated 184,052 HIV-infected children in Kenya, and 117,000 of them urgently need highly active antiretroviral therapy (HAART), yet only 24% have received HAART [6]. The urgency of reaching these remaining children is demonstrated by studies showing that 50% of children born with HIV will die before their second birthday if left untreated [7-9].

Targeted strategies to reach and identify untested individuals are critically needed to curb the HIV epidemic in Kenya. The family unit is one important portal. For each HIV-positive "index patient", one or more family members may be HIV positive or at high risk of HIV acquisition. Family members at risk in this context include sexual partners of index patients and the index patients' children younger than 15 years with HIV vulnerability stemming from sexual contact and mother to child transmission, respectively. Reaching these vulnerable family members begins with disclosure of one's HIV status. Disclosure to partners facilitates discussions on HIV and raises partner awareness about their risk and need to test [4]. Disclosure also has important health benefits. It increases access to social support, fosters closer relationships with others, increases testing uptake, improves treatment adherence and retention, and reduces risk of HIV transmission to partners [4,5,10]. This prevention potential is considerable among couples: in Kenya, 45% of HIV-infected married people have HIV-negative partners [1].

Although disclosure brings many benefits, there are significant obstacles and risks involved. Fear of negative outcomes is the most common barrier to women disclosing, and women who disclose risk violence from a partner if appropriate support is not present [4,5,10]. A study in Kenya found that 28% of women feared rejection by their family if they disclosed and 32% feared it would lead to partner break up [4]. However, a study of disclosure findings from 15 studies (14 in sub-Saharan Africa, including three in Kenya) found that actual disclosure outcomes were far more positive than women anticipated: the majority of women received supportive reactions after disclosing [5].

One of the studies in Kenya reported that 94% of HIV-positive women feared their partners' reactions, yet among women who disclosed to partners, 73% reported that partners were understanding [5]. Another study in Tanzania reported that 92% of women who disclosed remained in relationships that were intact [5]. The reason for this level of acceptance and support is not completely known; perhaps women had or were equipped with skills for safe disclosure.

This is not to say that negative outcomes are not experienced. In a study conducted in Kenya among women who disclosed, 3.5% reported being physically assaulted and 3.5% were chased from their homes, while in Tanzania, 15% experienced violence from their partners [5]. Preventing negative and harmful consequences is critical to patient and family well-being. Unfortunately, gender-based violence in Kenya and other sub-Saharan African settings has not been well addressed by HIV programmes [10].

Once vulnerable family members are reached for HIV testing, the opportunity to immediately enrol those who are HIV positive into care is created. Patients who enter HIV care early, before developing symptomatic disease, have better outcomes [11]. Family member testing also creates the opportunity to engage the family in care and support for those who are HIV positive. If one family member is HIV infected, the entire family is affected and has to cope with the physical, emotional, social and economic consequences of HIV. The family can be an important source of support. Studies in sub-Saharan Africa have found that the support of the family contributes to healthy behaviours and that partner involvement is associated with positive outcomes for HIV-infected member(s) [2,4,5,10,12-14].

To meet the challenges of reaching undetected and untreated people with HIV and to meet the psychosocial needs of infected and affected individuals, Family AIDS Care and Education Services (FACES), a programme working with the Ministry of Health to increase access to quality HIV services in Nyanza Province, decided to strategically draw on the strength of Kenyan family bonds to reach and treat vulnerable family members, reduce the risk of new infections, and garner family support for HIV-infected family members.

This study sought to describe the family model of care, as well as evaluate if this model is a viable way of identifying and enrolling family members into HIV care and treatment.

## Methods

### Family model of care

**Figure 1:**
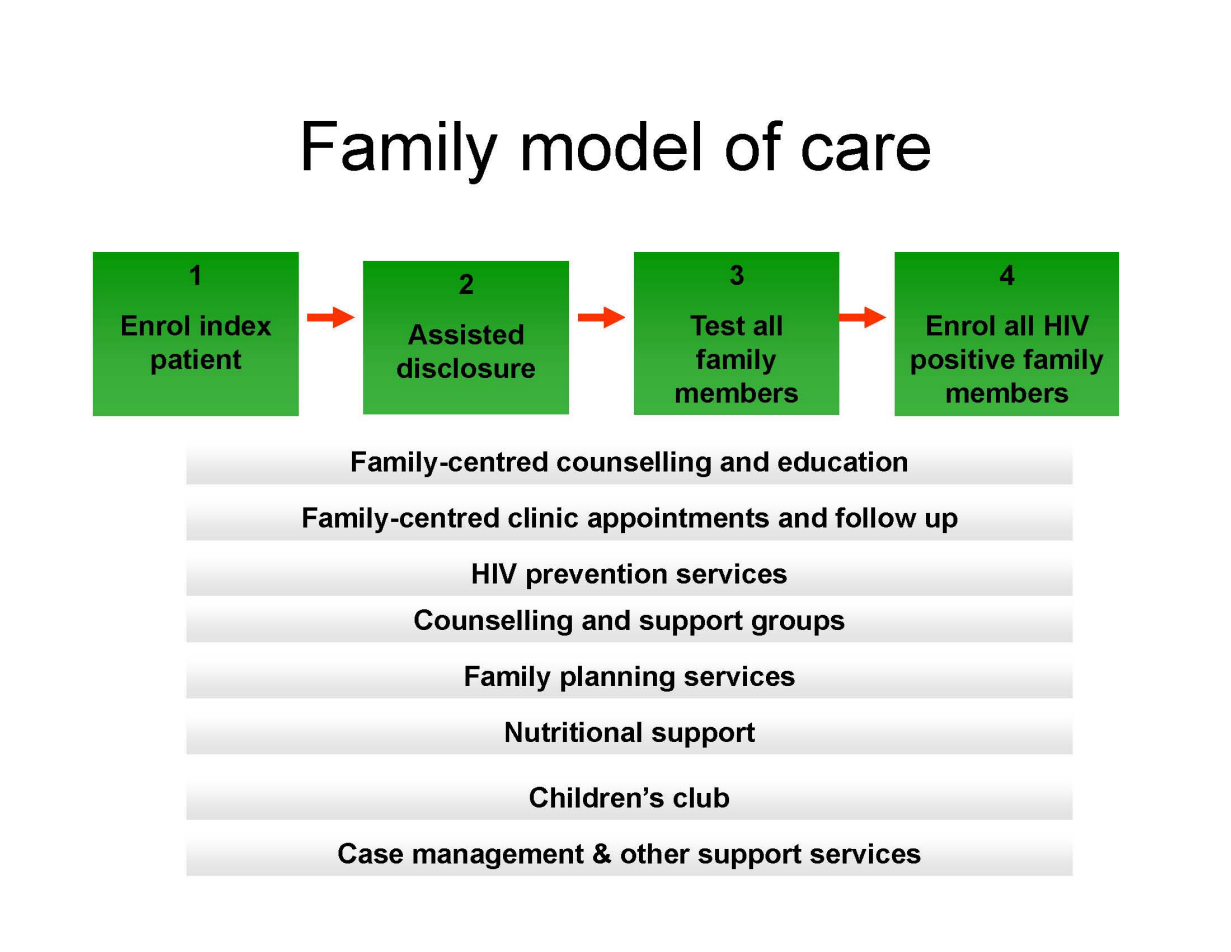
**Family model of care**. This diagram illustrates the family model of care approach. It is based on the linkage between index patients and their family members at risk. Index patients are enrolled, assisted disclosure is provided, vulnerable family members are identified and encouraged to come in for HIV testing, HIV-positive members are enrolled, prevention infection counselling is carried out for HIV-negative members, and family support and awareness is raised. Comprehensive services are designed to support the family unit.

The family model of care is based on the linkage between index patients and their family members at risk. A "family", in this context, is defined as two or more individuals who identify themselves as partners or family members, and "family members at risk" are defined as partners and/or children under 15 years of age of index patients. The family model of care is designed to identify, engage and care for all HIV-positive family members, prevent new infections among family members at risk, and raise family support and awareness within the HIV department at a health facility (Figure 1). Comprehensive family-centred services are built around this process.

### Identifying family members at risk

Foremost to the family-centred approach is identification of individuals at risk of HIV. Recognizing that each new patient is part of a family at risk for HIV, a system was developed to facilitate HIV testing among family members. On-site HIV education led by trained peer educators, who share their personal stories, is conducted to help patients gain a better understanding of the implications of HIV and how to live positively, including the importance of disclosure and family testing, as well discussing their personal barriers to disclosure.

During each clinical encounter, using a tool called the family information table (FIT), lay healthcare workers (HCWs) and providers guide patients through the steps of identifying family members at risk, assisted disclosure, bringing in family members for testing, enrolling those who are HIV positive into care, working with those who are HIV negative and HIV positive to prevent transmission, and building family support, including the encouragement of partner involvement. During subsequent clinic visits, providers update the FIT to continue to develop strategies with patients to overcome barriers to each of these steps.

### Assisted disclosure

Often the first barrier to family member testing is disclosure of HIV status due to stigma, denial and fear of rejection. To overcome the barrier of patients disclosing their HIV status to family members and to work towards achieving positive reactions, trained lay HCWs or nurses counsel patients through the disclosure process. Assisted disclosure begins with counselling on the risks and benefits of disclosure, followed by creating a personalized disclosure plan, including who the patients want to disclose to, in what order, and when, where and how to do it. Counsellors work to enhance patients' ability to disclose safely and to communicate with their partners about HIV. This sometimes involves role playing with the counsellor. Patients can decide to disclose on their own or in the presence of staff either at the clinic or in the patient's home or they can request a staff member to disclose on their behalf. If gender-based violence is suspected as a potential consequence, psychosocial counselling is provided, including discussion on alternative plans to disclosure.

### Testing family members at risk

Family members who are brought to the facility for testing are seen by nurses in a private setting. The nurses follow national guidelines for HIV counselling and testing, using the provider-initiated testing and counselling (PITC) approach. PITC involves providers taking the initiative to test patients for HIV at their point of care within a health facility. Family member testing is essentially an extension of PITC to include partners and family members of patients. Nurses review the purpose of the clinic visit, clarify their role as a counsellor, and determine previous testing status. They also work with the patients to determine if couples, family or individual testing is appropriate. Patient confidentiality is assured, and the nurses work to establish patient understanding of HIV. Consent follows the opt-out approach.

The HIV testing algorithm involves a rapid HIV antibody test. A second rapid test from a different manufacturer is performed if the first result is positive, and a third confirmatory test is conducted when the first two results are discordant. Infants younger than 18 months undergo an early infant diagnosis algorithm with DNA polymerase chain reaction (PCR) testing at six weeks of age. Those negative at six weeks of age undergo serial antibody testing at nine and 18 months. Positive antibody tests for any infants younger than 18 months are confirmed with DNA PCR testing. Patients with negative results are encouraged to return for repeat testing in three months' time, are counselled and referred for HIV prevention, and encouraged to be involved in their partners' HIV care. Patients with positive results receive post-test counselling and are encouraged to enrol into care. Testing outcomes are documented in the index patient's FIT.

### Enrolment into clinical care and supportive services

To facilitate family care and involvement, family members identified as HIV positive are immediately offered enrolment into care in a programme designed to support the family unit. Family members are booked for joint appointments, their files are kept together, couples and family counselling is provided, and family treatment buddies and partner involvement are encouraged. Family-friendly clinical services consist of opportunistic infection prevention and treatment, including tuberculosis and sexually transmitted infections, provision of highly active antiretroviral therapy, family case management, family planning, pre-conception counselling, nutritional support, and prevention with positives interventions.

Additional services include patient support groups to provide psychosocial support, build alliances, boost adherence, offer an open and accepting forum for discussion, and foster creative ideas and activities to strengthen families, including income-generation projects. It is common for HIV-infected children to have lost one or both parents and to be living with a single parent, grandparent or other guardian [15]. Therefore, ensuring that children have the best opportunity for quality care is a priority. Expert paediatric clinical care and counselling, a children's club, a caregiver support group, a children's breakfast programme and a waiting-area playground were instituted. Partnerships with local agencies were established for home-based care and educational support for children. Within antenatal care services, prevention of parent to child HIV transmission, integrated HIV services, early infant diagnosis for HIV-exposed children and partner testing are emphasized.

To optimize human resources and conserve valuable nurse and clinical officer time, a task-shifting approach is utilized to implement the family model of care. Lay HCWs, including peer educators and/or persons living with HIV, take on the non-clinical activities: trained lay HCWs conduct HIV education, provide disclosure support and counselling, complete the initial FIT, manage family files, and facilitate and/or support the children's, caregiver and patient support group activities. Nurses carry out family member testing and nurses and clinical officers are responsible for reviewing, updating, and guiding patients through the FIT during each clinical visit, enrolling family members into care, conducting couples and family counselling, and providing family-centred clinical services.

### Family information table evaluation methods

The family model of care approach was implemented in its earliest form at Lumumba Health Centre in Kisumu when HIV services were first launched in 2005. It was enhanced and refined over the next several years. An electronic medical record system (EMR) was implemented in September 2007 for patient enrolment records; however, the FIT was not integrated in or linked to the EMR. To evaluate the family model of care, a retrospective cross-sectional study was conducted among a sample of adult patients (15 years and older) seen between September 2007 and September 2009 at Lumumba Health Centre in Kisumu, Kenya.

The FITs were reviewed to assess HIV-infected index patients and family member linkages. Using a retrospective approach, a list of active adult patients between September 2007 and September 2009 was generated from the EMR system. There were 5802 active adult patients, including 1874 males and 3928 females. Among each gender cohort of patients, 5% (n = 96 males; n = 201 females; n = 297 total) were selected as the number of index patient FIT charts to audit. This sample size was chosen as it was a feasible number of FIT charts to audit and large enough to generate meaningful findings. Statistical Product and Service Solutions (SPSS) was then used to randomly select patients.

Charts were pulled for examination; 87 male charts and 198 female charts were reviewed, and three male charts and nine female charts were missing at the time of chart abstraction. This sample was used to determine the mean number of family members at risk (partners and children 0-14 years), proportion tested, proportion HIV positive, and proportion enrolled in care. Ninety-five percent confidence intervals (CIs) for proportions were estimated assuming an exact binomial distribution and 95% CIs for means were estimated assuming a Student's T distribution. Descriptive FIT data were analyzed in SPSS.

### Ethical review

Ethical review committee (ERC) permission was obtained locally and internationally; the protocol was reviewed for human subject concerns and approved by the Kenya Medical Research Institute ERC and University of California San Francisco Committee on Human Research.

## Results

Among 5802 adult patients, FIT data from a random sample of 285 patients were examined, including 87 (31%) male charts and 198 (69%) female charts. As shown in Table 1, these 285 patients led to the identification of 725 family members at risk of HIV (2.5 family members identified per index patient), including 241 (33%) partners and 484 (67%) children. Among family members at risk, 452 (62%) were tested for HIV (1.6 family members tested per index patient) of whom 175 (39%) were HIV positive. Of the 452 family members tested, 176 (39%) were partners and 276 (61%) were children. Among partners identified, 176 (73%) were tested, with 125 (71%) of them HIV positive, and among children identified, 276 (57%) were tested, with 50 (18%) of them HIV positive. Among HIV positive family members, 154 (88%) were enrolled in care, including 111 (89%) partners of HIV-positive partners and 43 (86%) children of HIV-positive children.

**Table 1:** Identification, HIV testing and enrolment into care of family members through a family-focused approach

	Index male		Index female		Index total	
	
	n (%) or mean	95% CI**	n (%) or mean	95% CI**	n (%)or mean	95% CI**
Sample size	96		201		297	

Missing	9		3		12	
**Sample**	**87 (31%)**		**198 (69%)**		**285**	
Measures						
Family members identified	236		489		725	
partners identified^†^	77 (33%)	(27%, 39%)	164 (34%)	(29%, 38%)	241 (33%)	(30%, 37%)
children identified^†^	159 (67%)	(61%, 73%)	325 (66%)	(62%, 71%)	484 (67%)	(63%, 70%)
Family members tested for HIV^†^	137 (58%)	(51%, 64%)	315 (64%)	(60%, 69%)	452 (62%)	(59%, 66%)
partners tested for HIV*	63 (82%)	(71%, 90%)	113 (69%)	(61%, 76%)	176 (73%)	(67%, 79%)
children tested for HIV*	74 (47%)	(39%, 55%)	202 (62%)	(57%, 67%)	276 (57%)	(52%, 61%)
Family members HIV positive*	52 (38%)	(30%, 47%)	123 (39%)	(33%, 45%)	175 (39%)	(34%, 43%)
partners HIV positive^‡^	41 (65%)	(52%, 77%)	84 (74%)	(65%, 82%)	125 (71%)	(64%, 78%)
children HIV positive ^‡^	11 (15%)	(8%, 25%)	39 (19%)	(14%, 25%)	50 (18%)	(14%, 23%)
Family members enrolled^¥^	47 (90%)	(79%, 97%)	107 (87%)	(80%, 92%)	154 (88%)	(82%, 92%)
partners enrolled^¥¥^	39 (95%)	(83%, 99%)	72 (86%)	(76%, 92%)	111 (89%)	(82%, 94%)
children enrolled ^¥¥^	8 (73%)	(39%, 94%)	35 (90%)	(76%, 97%)	43 (86%)	(73%, 94%)

Family member identification per index patient	2.71	(2.33, 3.09)	2.47	(2.25, 2.68)	2.54	(2.36, 2.73)

Family member tests per index patient ^▶▶^	1.57	(1.26, 1.89)	1.59	(1.40, 1.79)	1.59	(1.42, 1.75)

^† denominator is "family members identified"^

* ^denominators are "partners identified" and "children identified"^

^‡ denominators are "partners tested for HIV" and "children tested for HIV"^

^¥ denominator is "family member HIV positive"^

^¥¥ denominators are "partners HIV positive" and "children HIV positive"^

^▶"family member reach per index patient" was calculated by taking total reached and dividing by the index N^

^▶▶family member tests per index patient was calculated by taking total tested and dividing by the index N^

** ^Confidence intervals around proportions were calculated using binomial exact distribution; confidence intervals for means calculated were based on Student's T distribution.^

## Discussion

As a governing programme strategy, this family model of care approach is used to facilitate patient identification, testing and subsequent enrolment into care in a practical manner; it optimizes interactions with index patients during routine HIV care visits. Providers view each index patient as a link to family members who could be in need of HIV care or prevention services. The family model of care also provides a framework to support patients and families through comprehensive family-friendly services.

Without the push to identify family members in need of HIV testing, the programme would rely on voluntary counselling and testing and other department and facility referrals. However, through family member testing, for each index patient, approximately 2.5 family members at risk were identified and about 1.6 family members were tested. It was particularly instrumental in reaching children; 61% of family members identified and tested through the FIT were children. Similar findings were reported in a PMTCT family-centred study: two-thirds of women enrolled in PMTCT enrolled a family member, primarily their HIV-exposed infants [9]. Family testing captured in the FIT also revealed a high proportion of HIV-positive partners (71%) and high enrolment uptake (89%) among HIV-positive partners. Family member referrals may also have the advantage of advancing enrolment for HIV-infected individuals who may have otherwise waited until they were symptomatic to be tested. This has a clear advantage to patients since later enrolment is associated with poorer health outcomes [11].

Limitations to this data include the absence of a link between testing data and EMR encounter data, a relatively small sample selection of the FIT, limited documentation in the FIT, and the possibility that additional HIV positive family members may have enrolled after the timeframe examined. Patient identifiers that facilitate linkage between testing and enrolment data are needed to better track patients across testing and care services.

The FIT serves as an important tool for providers. It prompts and facilitates ongoing follow up with patients regarding their family disclosure, testing and enrolment status in a systematic manner. The FIT could be even more beneficial if incorporated into the electronic medical records system with variables that link testing results and enrolment status more fluidly.

The clinical and support services offered through the family model of care are tailored to break down barriers to disclosure, testing and care and to garner family support for patients, although this was not formally evaluated. Other studies corroborate that strong family bonds and partner engagement in care bolster health outcomes and benefit HIV-affected family members [4,5,9]. Nevertheless, continued attention and novel interventions are needed to further increase disclosure, build family support, and improve partner and child testing and enrolment into HIV care.

## Conclusions

The family model of care is a feasible approach to broaden HIV case detection and service reach. The approach can be adapted for the local context and should continue to optimize index patient linkages, FIT adaption and usage, and innovative methods to increase family testing and to package services for families in a manner that builds on family support and enhances patient care and prevention efforts. Further efforts are needed to increase family member engagement.

## Competing interests

The authors declare that they have no competing interests.

## Authors' contributions

JLK participated in the evaluation design and took the lead in drafting the manuscript. JAP participated in the programme and evaluation design and provided technical expertise on manuscript review, coordination and editing. RM helped draft and edit the manuscript. PO provided programme technical expertise. AOO oversaw data collection and conducted statistical analysis. SBS provided technical review of the evaluation design and data analysis. CCB provided statistical analysis consultation. LN helped draft and review the manuscript. CWM helped draft and review the manuscript. HCM provided a technical review of the draft manuscript. EAB participated in the programme and evaluation design and helped draft the manuscript. CRC conceived the programme and the study and helped draft and review the manuscript. All authors read and approved the final version of this manuscript.
